# Platelet Activation Mechanisms and Consequences of Immune Thrombocytopenia

**DOI:** 10.3390/cells10123386

**Published:** 2021-12-01

**Authors:** Siyu Sun, Rolf T. Urbanus, Hugo ten Cate, Philip G. de Groot, Bas de Laat, Johan W. M. Heemskerk, Mark Roest

**Affiliations:** 1 Department of Biochemistry, Cardiovascular Research Institute Maastricht (CARIM), Maastricht University, 6200 MD Maastricht, The Netherlands; s.sun@thrombin.com (S.S.); h.tencate@maastrichtuniversity.nl (H.t.C.); 2 Synapse Research Institute, 6217 KD Maastricht, The Netherlands; ph.degroot@thrombin.com (P.G.d.G.); b.delaat@thrombin.com (B.d.L.); 3 Center for Benign Haematology, Thrombosis and Haemostasis, Van Creveldkliniek, University Medical Center Utrecht, Utrecht University, 3584 CX Utrecht, The Netherlands; R.T.Urbanus@umcutrecht.nl; 4 Maastricht University Medical Center, Department of Internal Medicine, Cardiovascular Research Institute Maastricht (CARIM), Maastricht University, 6200 MD Maastricht, The Netherlands

**Keywords:** autoimmune disorders, immune thrombocytopenia, platelet, thrombosis, auto-antibodies

## Abstract

Autoimmune disorders are often associated with low platelet count or thrombocytopenia. In immune-induced thrombocytopenia (IIT), a common mechanism is increased platelet activity, which can have an increased risk of thrombosis. In addition, or alternatively, auto-antibodies suppress platelet formation or augment platelet clearance. Effects of the auto-antibodies are linked to the unique structural and functional characteristics of platelets. Conversely, prior platelet activation may contribute to the innate and adaptive immune responses. Extensive interplay between platelets, coagulation and complement activation processes may aggravate the pathology. Here, we present an overview of the reported molecular causes and consequences of IIT in the most common forms of autoimmune disorders. These include idiopathic thrombocytopenic purpura (ITP), systemic lupus erythematosus (SLE), antiphospholipid syndrome (APS), drug-induced thrombocytopenia (DITP), heparin-induced thrombocytopenia (HIT), COVID-19 vaccine-induced thrombosis with thrombocytopenia (VITT), thrombotic thrombocytopenia purpura (TTP), and hemolysis, the elevated liver enzymes and low platelet (HELLP) syndrome. We focus on the platelet receptors that bind auto-antibodies, the immune complexes, damage-associated molecular patterns (DAMPs) and complement factors. In addition, we review how circulating platelets serve as a reservoir of immunomodulatory molecules. By this update on the molecular mechanisms and the roles of platelets in the pathogenesis of autoimmune diseases, we highlight platelet-based pathways that can predispose for thrombocytopenia and are linked thrombotic or bleeding events.

## 1. Introduction

Platelets are abundantly circulating anucleate blood cells that are produced from mature megakaryocytes, predominantly in the bone marrow. Thrombopoietin, synthesized in the liver, acts as a central regulator of platelet production via stimulation of the megakaryocytic MPL receptors [1]. Under physiological conditions, the lifespan of the formed platelets is approximately 7–10 days, but this time can greatly be shortened in pathologic conditions, for instance due to platelet activation or by enhanced platelet destruction. In healthy subjects, the number of circulating platelets is known to be determined by the rate of platelet formation and the removal rate of aging platelets from the circulation [2,3].

Platelets are crucial not only in hemostasis and thrombosis, but also in immune reactions. In the non-diseased blood vessels, the endothelium continuously prevents platelets from activation and aggregation through a variety of mechanisms. These are provided by extracellular ectonucleotidases, by expression of thrombin-inhibiting anticoagulants (thrombomodulin and heparan sulfates), and by secreted nitric oxide and prostacyclin (prostaglandin I_2_) [4]. At sites of vascular injury or activation, this suppression becomes impaired, which can result in an enhanced platelet adhesion to the vessel wall via endothelial-released von Willebrand factor (VWF), chemokines and other mediators [5,6]. In an atherosclerotic vessel, local endothelial inflammation may thus lead to a chronic proinflammatory and procoagulant state, causing increased leukocyte attraction and platelet adhesion even in the absence of endothelial injury [4].

Endothelial injury acutely triggers to thrombus formation, in that circulating platelets rapidly interact with exposed subendothelial collagen and VWF through the receptors glycoprotein VI (GPVI), integrin α2β1 and glycoprotein Ib-V-IX (GPIb-V-IX) [7]. In addition, the coagulation process is triggered by tissue factor (extrinsic pathway) and factor XII (intrinsic pathway), resulting in sudden thrombin and fibrin generation [8,9]. The formed thrombin also stimulates human platelets via the protease-activated receptors PAR1 and PAR4. Collagen and thrombin stimulate the exposure of procoagulant phosphatidylserine at the platelet surface, which promotes the assembly of coagulation factors and potentiates the formation of thrombin and fibrin [10]. Activated platelets release autocrine mediators to augment the activation of nearly, which include thromboxane A_2_, binding to the thromboxane-prostanoid receptors (TP); and the δ-granule product ADP, acting via the purinergic receptors (P2Y_1_, P2Y_12_), as well as ATP, operating via P2X_1_ receptors [11,12]. Platelet aggregates are built by conformation changes of the abundantly expressed integrin αIIbβ3 on the platelet membrane, allowing interaction with plasma fibrinogen, a bivalent protein than bridges integrins between adjacent platelets [7].

For a long time, it was thought that the same or a similar set of molecules and receptors regulate platelet activation in hemostasis and in diverse pathological conditions, including arterial and venous thrombus formation, thrombo-inflammation and immune responses [7]. The ability of platelet aggregate formation in normal hemostasis requires the presence of relatively high platelet counts in the blood. This has been formalized by establishing normal reference ranges, below which one speaks of thrombocytopenia. According to a common definition, this is at <150 × 10^9^ platelets/L [13]. However, it appears that in healthy subjects the hemostatic process remains non-affected until the platelet count drops down to <30 × 10^9^/L. In the case of pathological thrombocytopenia, unwanted bleeds can occur after trauma or surgery, although the severity of the bleeding disorder can vary from person to person [13]. Spontaneous bleeds are observed at even lower platelet counts of <10 × 10^9^/L. On the other hand, in cases of genetic or immune conditions of thrombocytopenia, an undesired activation or dysfunction of platelets can result in bleeding and/or thrombosis at substantially higher platelet counts than 30 × 10^9^/L [14]. In the condition of immune-driven thrombocytopenia, platelets can be activated or destroyed in a pathological manner by pathways that differ from those in normal hemostasis [15].

**Figure 1:**
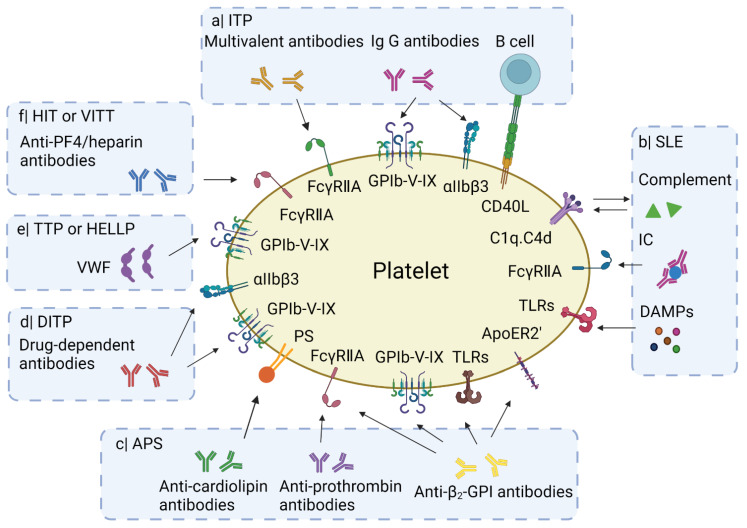
Overview of mechanisms leading to platelet activation in types of IIT. a| Frequently in ITP, IgG auto-antibodies bind to GPIbα or αIIbβ3 on the platelet membrane. Incidentally multivalent auto-antibodies induce platelet activation via FcγRIIA receptors. The glycoprotein CD40L (CD154) on activated platelets can interact with B cell CD40, inducing B cell proliferation. b| In SLE, immune complex (IC) and DAMPs (e.g., HMGB1 or S100A8/9) activate platelets through binding to surface receptors such as FcγRIIA and Toll-like receptors (TLRs). The IC may also activate the complement system leading to deposition of complement fragments (C1q, C4d) on the platelet surface, which potentiates their activation. c| Anti-cardiolipin, anti-β_2_-GPI and anti-prothrombin antibodies can induce platelet activation in APS patients. d| In TTP or HELLP, ultra-large VWF multimers provoke the agglutination of platelets via GPIbα. e| In HIT or VITT, antibodies to PF4/heparin complexes bind and activate platelets via FcγRIIA receptors. For further explanations, see text. Created with Biorender.com.

**Figure 2:**
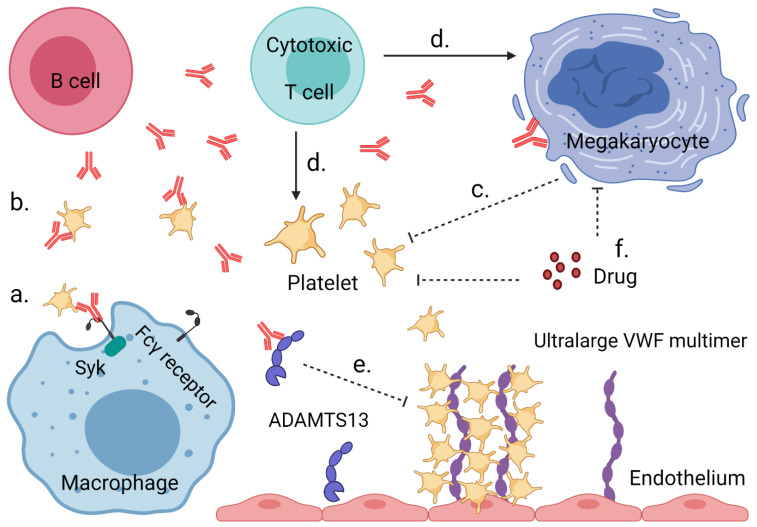
Mechanisms leading to low platelet count or thrombocytopenia in IIT. a| Auto-antibodies target platelets for destruction by macrophages in the spleen or liver through Fcγ receptor signaling via spleen tyrosine kinase (Syk). b| Platelets decorated with auto-antibodies can also be destroyed via other mechanisms, such as complement activation or desialylation. c| Auto-antibodies binding to megakaryocytes can lead to an impaired platelet production. d| Cytotoxic T cells can directly destroy or inhibit platelets and megakaryocytes. e| Binding of ultra-large VWF multimers to platelets induces microthrombus formation in the absence of functional ADAMTS13 (either inhibited by auto-antibodies or not expressed due to a congenital defect). f| Chemotherapeutic antiproliferative drugs can suppress megakaryocyte development and platelet production. Created with Biorender.com.

In the present review, we summarize reported key mechanisms of platelet activation (Figure 1), in particular focusing on idiopathic thrombocytopenic purpura (ITP), systemic lupus erythematosus (SLE), antiphospholipid syndrome (APS), drug-induced thrombocytopenia (DITP), heparin-induced thrombocytopenia (HIT), thrombotic thrombocytopenia purpura (TTP), hemolysis, elevated liver enzymes and low platelets (HELLP) syndrome, and COVID-19 vaccine-induced thrombosis with thrombocytopenia (VITT). In addition, we list the reported mechanisms of platelet destruction in these immune disorders (Figure 2). For each pathology, we also present information on disease type, description, bleeding vs. thrombosis and implicated genes in a tabular form (Supplementary Table S1). We furthermore compiled information on the clinical phenotype and symptoms, blood abnormalities, treatment options, and platelet phenotypes (Supplementary Table S2).

## 2. Platelets in Idiopathic Thrombocytopenic Purpura (ITP)

Idiopathic thrombocytopenic purpura (ITP, also known as immune thrombocytopenia or immune thrombocytopenic purpura) is commonly defined as an isolated thrombocytopenic autoimmune disorder without known underlying cause and is diagnosed when the platelet count is <100 × 10^9^/L. Many of the ITP patients report a reduced health-related quality of life, in which fatigue is quite common [16]. Patients can also be asymptomatic or experience bruise-like rashes or mild mucocutaneous bleeds. Severe bleeds are reported in only 5% of the patients. Despite low platelet counts, the risk of venous thromboembolism is twice higher in ITP patients than in the general population [17].

The pathogenesis of low platelet count or thrombocytopenia may be due to either a low production of platelets or a high removal of platelets from the peripheral circulation [18]. Platelet-exposed CD40L (CD154) can drive the activation of autoreactive B lymphocytes in ITP, inducing the production of auto-antibodies [19]. The proven production of auto-antibodies, particularly the class of immunoglobulin G (IgG), is considered to be a pathogenic indication of ITP [15,20]. On the other hand, failure to detect such antibodies does not exclude ITP. Several mechanisms contribute to ITP, including cytotoxic T cell-mediated platelet destruction and megakaryocytes inhibition, microthrombus formation due to platelet activation by auto-antibodies, phagocytosis of antibody-opsonized platelets, enhanced platelet desialylation and apoptosis, and complement activation [21,22].

**Figure 3:**
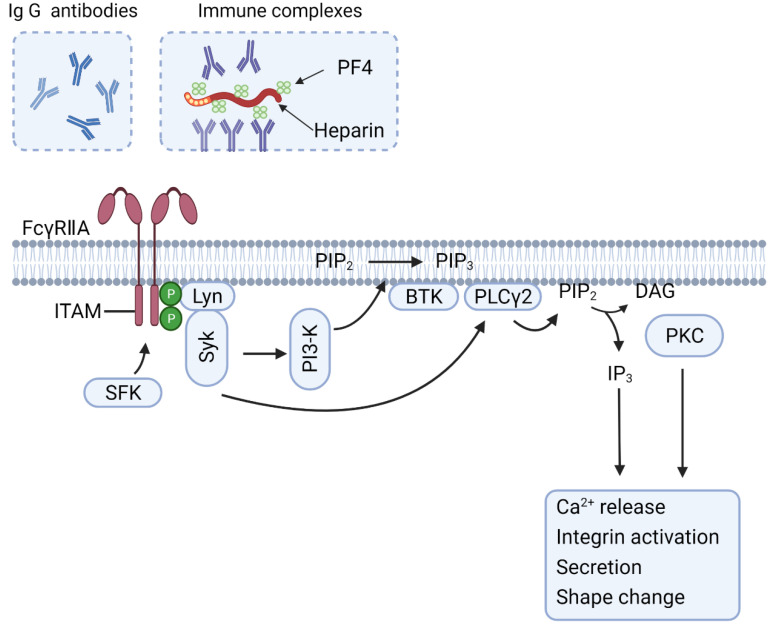
FcγRIIA-induced platelet activation and signaling pathways. IgG antibodies or larger immune complexes can activate platelets by dimerization or clustering of FcγRIIA receptors. The tyrosine-based activation motif (ITAM) is then phosphorylated by Src-family kinases (SFK), recruiting and phosphorylating the tyrosine kinase Syk, which in turn activates phosphoinositide 3-kinase (PI3K). The production of phosphatidylinositol-3,4,5-trisphosphate (PIP_3_) recruits Bruton’s tyrosine kinase (BTK) and phospholipase Cγ (PLCγ), which leads to activation of downstream responses via IP_3_ production and protein kinase C (PKC). The extent of platelet activation is likely dependent on the type of IgG antibodies and immune complexes. Created with BioRender.com.

Antiplatelet antibodies are detected in 50–60% of the ITP patients [23]. These auto-antibodies are frequently directed against abundantly present glycoproteins at the platelet surface, particularly GPIb-V-IX and integrin αIIbβ3, and more rarely integrin α2β1 [24]. Auto-antibody-opsonized platelets have been identified in macrophages in the liver, spleen or both with an absorbance role of receptors of the Fcγ class [18,25]. In the macrophages, especially the low affinity receptors FcγRIIA and FcγRIIIA, both transmitting signals through an ITAM (immuno-receptor tyrosine-based activation motif), seem to be responsible for the clearance of opsonized platelets [26,27]. In a small subgroup of patients with antibodies targeting the GPIb-V-IX complex, the binding of multivalent autoantibodies also engages the platelet FcγRIIA receptors [28], which induces signaling via ITAM phosphorylation and activation of the tyrosine kinase Syk, which downstream results in the increase of intracellular calcium, integrin activation, secretion and the shape change (Figure 3) [29]. It is suggested that the clearance of FcγRIIA-activated platelets is mediated by the surface expression of P-selectin and phosphatidylserine, which are all ‘eat-me’ signals for macrophages [28]. Future studies need to prove this.

In patients with chronic ITP, the long-term circulating auto-antibodies may affect the differentiation and migration of megakaryocytes in the bone marrow, with as a consequence low proplatelet formation, while the proliferation and viability of megakaryocytes remain unchanged [30]. Since the polyploid megakaryocytes express both GPIb-V-IX and αIIbβ3, also these bone marrow cells can provide a target surface for antiplatelet auto-antibodies [31,32]. Whether FcγRIIA-mediated megakaryocytes activation affects platelet production has not been investigated.

In murine models of ITP, it was demonstrated that the intravenous injection of anti-integrin αIIb antibodies caused platelet apoptosis with ensuing thrombocytopenia. Subsequent administration of immunoglobulins inhibited the apoptotic process, as was concluded from the reduction in phosphatidylserine exposure and caspase-3 activation. The immunoglobulin intervention also diminished the thrombocytopenia [33]. In agreement with this work, also in pediatric and adult patients with acute ITP, the infusion of immunoglobulins could suppress platelet apoptosis and increased the platelet count [34]. It is worth noting that such enhanced platelet apoptosis is especially observed in ITP patients carrying anti-GPIb-V-IX (anti-GPIbα) or anti-αIIbβ3 antibodies, but not in subjects with anti-α2β1 antibodies (binding to the fewer copies of integrin α2β1) [35]. This points to an auto-antibody-specific FcγRIIA-induced platelet activation, perhaps depending on the abundance of the primary immune target.

As an alternative, Fc-independent platelet clearance pathway, the desialylation of platelet surface glycoproteins has been used as diagnostic biomarker and possible therapeutic target for refractory forms of ITP [36]. Both in vivo mouse studies and observations of ITP patients indicate that anti-GPIbα auto-antibodies induce opsonization of platelets lacking sialic acid residues. This can result in platelet clearance by hepatocytes via the Ashwell-Morell receptors [37]. In this setting, a positive feedback loop has been proposed of platelet desialylation and platelet activation, in which the binding of anti-GPIbα auto-antibodies initiates neuraminidase-1 release and subsequent desialylation of GPIbα. This externalization of neuraminidase-1, cleaving the terminal sialic residues of GPIbα and other glycoproteins, then fosters receptor clustering and therefore amplifies the platelet activation process [38]. From experiments with mice containing a hematopoietic cell-specific defect in O-linked glycosylation, it was concluded that desialylated platelets are cleared by the hepatic Kupffer cells via the Ashwell-Morell receptors in conjunction with CLEC4F receptors [39,40]. However, the importance of this clearance pathway still needs to be verified in ITP patients.

Recently, it was proposed that anti-GPIbα antibodies can evoke a platelet clearance pathway that acts independently of Fc receptors [41]. Bivalent anti-GPIbα antibodies then bind the GPIbα chains on adjacent platelets, thereby promoting an agglutination process. Under conditions of high shear flow, such platelet cross-linking can unfold the juxta-membrane mechanosensory domains of GPIbα, which may also drive the clearance of platelets. Interestingly, the binding of VWF to GPIbα, which also unfolds the mechanosensory domain, appeared to cause neuraminidase-1 release and subsequent desialylation of the platelets [42].

In some ITP patients, antiplatelet auto-antibodies appeared to trigger the classical complement pathway [43]. Platelet clearance then was induced through complement-induced cellular lysis or through factor C3b-mediated phagocytosis [24]. Additionally, the complement-fixing auto-antibodies were found to mostly target the abundant complexes GPIb-V-IX and αIIbβ3 [43]. An early report stated that antibody-mediated complement fixation also caused the shedding from platelets of extracellular vesicles (microparticles) [44]. It was proposed that both complement activation by antibody-coated platelets and vesicle formation contributed to the pathogenesis of thrombosis in ITP [45].

Next to auto-antibody-mediated platelet destruction, also T cell-mediated cytotoxicity is hypothesized as a mechanism of ITP. Platelets that present autoantigens via the major histocompatibility complex class I molecules are susceptible to lysis via cytotoxic T-lymphocytes [46,47]. This implies that the blockage of the T-cell activation would reduce the platelet destruction in ITP, even for patients not carrying detectable auto-antibodies. More work needs to be done to test this hypothesis.

An uncommon form of ITP is a consequence of congenital immunodeficiency (congenital ITP), a condition that is usually linked to rare genetic defects affecting the functions of platelets as well as immune cells. Thus, patients with a dysfunctional mutation in the *ORAI1* or *STIM1* genes suffer from severe immunodeficiency, ectodermal dysplasia and other Ca^2+^-linked abnormalities, including a reduced platelet count and platelet signaling defects [48]. Similar congenital forms of ITP include gene defects in *ACP5*, *ARHGEF1*, *ARPC1B*, *CTLA4*, *LAT*, *RAB27A*, *STAT3*, *WAS* and *WDR1* (Supplementary Table S1). Of genes linked to supposedly non-congenital forms of ITP, only *FCGR2C* is mentioned, i.e., the Fc fragment of IgG receptor IIc. For an extensive overview of genes in inherited thrombocytopenia, we refer to another paper [49].

## 3. Platelets in Systemic Lupus Erythematosus (SLE)

SLE is characterized as an autoimmune disease associated with chronic inflammation and organ damage [50]. The pathogenesis of SLE comprises the dysregulation of lymphocyte functions and the production of auto-antibodies, particularly against nucleic acids. Thrombocytopenia is observed in 10–15% of the SLE patients [51]. Yet, the thrombocytopenia is relatively common in patients with severe manifestations of SLE, such as hemolytic anemia, neuropsychiatric symptoms and kidney injury [52]. In line with this, a low platelet count is regarded as a prognostic indicator of survival in SLE.

The currently accepted mechanism for thrombocytopenia in SLE patients is that platelets are destructed by antiplatelet auto-antibodies, in an analogous way as described for classical ITP. In addition to antiplatelet antibodies, patients may also present with antiphospholipid antibodies, which in turn bind to platelets [53]. This contrasts to the situation in ITP, where auto-antibodies are usually targeting GPIb-V-IX or integrin αIIbβ3. Hence, the mechanism of thrombocytopenia in SLE relies on the more complex interactions between antiphospholipid and platelet-antigen antibodies [50,54].

In a subset of patients with SLE, platelet production in the bone narrow is impaired or platelet sequestration in the spleen is increased, both of which processes that are contribute to the thrombocytopenia [54]. Additionally, high levels of IgG containing-immune complexes are found in many cases of SLE [55]. Such complexes can initiate platelet activation via the FcγRIIA receptors [56]. The FcγRIIA-induced platelet activation and signaling pathways are shown in Figure 3. In addition, the FcγRIIA-activated platelets via immune complexes release serotonin and can be temporarily sequestered in confined vascular beds, for instance in the leaky vasculature of brain and lungs [57]. The sequestered platelets may after degranulation return to the peripheral circulation. Other reported platelet pathways in SLE include activation by Toll-like receptors, complement activation, shedding of extracellular vesicles and ischemia-reperfusion associated with Raynaud phenomenon (a medical condition in which the spasm of small arteries causes episodes of reduced blood flow through fingers and toes) [50,58].

While immune complex-mediated platelet sequestration is considered an underlying mechanism of the thrombocytopenia in SLE, only few studies have registered the actual causes of low platelet counts. It is conceivable that other clinical parameters are relevant as well, implicating that the thrombocytopenia is due to a set of multiple and partly overlapping pathogenic mechanisms.

There is some evidence for a genetic basis of (resistance to) SLE, although the genetic setting is complex. Genome-wide screens and family studies point to associations with about 30 genes ( Supplementary Table S1), where the relation to thrombocytopenia is mostly unknown [59].

## 4. Platelets in Antiphospholipid Syndrome (APS)

APS is a condition, characterized by vascular thromboembolism or obstetric complications in combination with the persistent serological presence of antiphospholipid antibodies [60]. The so-called antiphospholipid antibodies are mostly targeted at plasmatic β2-glycoprotein I (β2-GPI) and prothrombin, proteins that avidly bind to procoagulant phospholipids. The presence of such antibodies is detected with three different types of assays: a phospholipid-dependent coagulation assay showing prolongation; an anti-cardiolipin IgG/IgM assay; or an anti-β_2_-GPI IgG/IgM assay [61]. Moreover, clinical classification criteria of thrombosis and/or pregnancy morbidity, patients with APS display a variety of other symptoms including thrombocytopenia and low white blood cell counts.

Because the thrombocytopenia is rarely severe, bleeding is not often seen and less common than thrombosis. Low platelet counts are thus regarded as a common ‘non-criterium’ hematologic manifestation, which occurs in 20–50% of the patients with confirmed APS [62]. On the other hand, a pronounced thrombocytopenia more often links to severe APS phenotypes, such as increased risk of thrombosis [63]. Although the pathophysiology of thrombocytopenia in APS is incompletely elucidated, several mechanisms have been put forward. These include antiphospholipid-mediated platelet activation and consumption, ITP-like auto-antibodies causing platelet destruction, and a thrombotic microangiopathy [64].

The concurrence of thrombosis and thrombocytopenia in APS patients points to platelets as major disease modifiers [54]. Platelet activation in relation to lupus anticoagulant (a subset of auto-antibodies that prolong the clotting time in a diagnostic test) has amply been investigated in the last two decades. It has been shown that antiphospholipid antibodies recognize proteins with high affinity for anionic phospholipids, such as on procoagulant platelets. Some receptors have been postulated to mediate platelet activation via co-factor-antibody complexes, such as Toll-like receptors, ApoER2′, GPIb-V-IX and FcγRIIA [65,66]. Whether phosphatidylserine exposure is needed before receptor binding is currently unclear. Interactions between anti-β_2_-GPI antibodies and the ApoER2′-GPIbα axis on platelets are known to sensitize platelets, but enhance platelet activation only with additional stimuli [66]. Nevertheless, direct platelet activation as a result of antiphospholipid antibodies through engagement of FcγRIIA receptors has also been reported [67,68]. It is conceivable that a continuous low-grade activation of platelets by the cofactor-antibody complexes results in a higher platelet turnover, ultimately resulting in thrombosis combined with thrombocytopenia. In line with this idea, a positive feedback loop between low platelet counts and the formation of platelet rich thrombi has been suggested [62].

## 5. Platelets in Drug-Induced Thrombocytopenia (DITP)

Several chemotherapeutics and immunosuppressive drugs can provoke thrombocytopenia by distinct mechanisms, ranging from the suppression of platelet production and triggering of platelet activation to enhancement of platelet destruction, as reviewed elsewhere [69]. Some of these drugs are capable of inducing auto-antibodies, either by drug binding to platelets (quinines) or after inducing platelet activation (integrin antagonists tirofiban, eptifibatide, abciximab). The antibodies induced by quinine isomers are known to be raised to abundant epitopes on the platelet surface, in particular integrin αIIbβ3 and GPIb-V-IX [70]. An indication for DITP is the detection of an IgG that binds to platelets in the presence of that drug (or a metabolite). The generation of drug-dependent antibodies is well-known for reversible integrin αIIbβ3 antagonists [70]. Antibodies can even be occurring without (known) prior drug exposure. Consequences of IgG binding, whether or not via FcγRIIA receptors, are platelet (in)activation, platelet sequestration in the spleen and other organs, and/or diminished platelet production [69,71].

## 6. Platelets in Heparin-Induced Thrombocytopenia (HIT)

In a minority of hospitalized patients, the administration of heparin induces thrombocytopenia, called HIT, as a potentially devastating immune-mediated drug reaction. Thrombocytopenia is especially seen after intraoperative heparin administration upon cardiac or vascular surgery, or postoperatively after the application of heparin as a thromboprophylaxis. Referred to as type I (heparin-associated thrombocytopenia, 10% of cases), HIT concerns a non-immunologic response to heparin, instigated by the interaction between heparin and circulating platelets, and resulting in platelet agglutination [72]. In this case, the thrombocytopenia is usually mild and transient, occurring within 2–3 days of treatment.

Alternatively, HIT type II typically develops at 5–14 days after exposure to heparin, and is then caused by antibodies, which are directed against the complex of heparin and platelet factor 4 (PF4), the latter being a platelet secretion product. As a soluble protein, PF4 is in dynamic equilibrium between monomers, dimers and tetramers [73]. The positively charged PF4-mers avidly bind to the negatively charged heparin molecules, leading to the formation of a growing combined complex. In a small subset of patients, conformational changes in the complex raise an immune response [74]. Herein, the exposure of neo-epitopes in the PF4-heparin complexes can induce IgG antibody generation and binding to platelets [74]. It was found that the presence of PF4 tetramers drives the formation of ultra-large PF4-heparin complexes, which also have the highest immunogenicity [75]. Presently, the presence in patients of anti-PF4 polyanion (even non-heparin) antibodies is considered an indication of autoimmune HIT [76]. There is evidence that the molecular size of the PF4-heparin complexes determines the ultimate platelet responses to HIT antibodies, with a stronger activation signal seen for ultra-large complexes [75,76]. This explains why only a subset of heparin-treated patients develops HIT and thrombosis (HITT). As a HIT-like syndrome, the vaccination-induced thrombocytopenia is elaborated in the VITT section.

In HIT, platelets are considered to become activated via antibodies to PF4/heparin complexes binding to the FcγRIIA receptors, which can lead to intravascular microthrombus formation and ultimately thrombosis [77,78]. Recent findings indicate that next to the FcγRIIA receptors also thrombin receptors can play a role. Downstream activation responses include secretion, platelet-leukocyte complex formation, procoagulant activity, and consequent necrotic- and apoptotic-like features [79,80]. Studies with transgenic mice expressing human FcγRIIA in the platelets have confirmed the role of ITAM-domain mediated signaling to Syk kinase in response to HIT antibodies (Figure 3) [81]. In addition, HIT antibodies can activate monocytes and (in)directly endothelial cells, inducing up-regulation of active tissue factor [82]. As a consequence, thrombin generation will be increased [82,83].

Regarding diagnosis, an otherwise unexplained low platelet count at 5–12 days after heparin treatment is an indication for type 2 HIT. The concerning clinical scoring system is based on 4Ts: thrombocytopenia, timing of platelet count fall, thrombosis or other sequelae, and lack of other explanations for thrombocytopenia [84]. Laboratory criteria to confirm HIT functionally include heparin-induced platelet activation tests and PF4-dependent immunoassays [85]. Other advanced immunoassays for HIT are based on the detection of antibodies binding to PF4/polyanion complexes using a variety of methods [83].

When HIT is suspected or confirmed, heparins need to be avoided and alternative anticoagulant treatment should be given [86]. In the management of acute HIT of a hospitalized patient, this means other parenteral thrombin-inhibiting agents (e.g., argatroban, bivaluridin, or danaparoid). In a recent meta-analysis, the application of argatroban appeared to associate with the lowest rates of thromboembolic events and of bleeding complications, on top of a shorter hospitalization length [87]. A systematic review concluded a similarity in the efficacy and safety of fondaparinux (heparin-derivative), bivalirudin or danaparoid [88].

## 7. Platelets in Vaccine-Induced Thrombosis with Thrombocytopenia (VITT)

Since the SARS-CoV-2 pandemic of 2020, and the fast development of vaccines against the virus, there has been worldwide interest in rare events of thrombotic thrombocytopenia, first identified in the cerebral venous sinus, after vaccination [89,90,91]. VITT is regarded as a new syndrome associated with preparations of adenoviral vector-based COVID-19 vaccines [90,92]. Recent findings point to a more prominent (1:100,000 cases) presentation after ChadOx1 nCoV-19 vaccination than after Ad.26COV.2.S vaccination, likely related to the composition of the vaccines [93,94]. Both systemic venous and arterial thrombotic complications have been reported. At the time of writing this paper, there is insufficient information on VITT by other than adenoviral vector-based vaccines for preventive treatment against SARS-CoV-2 infections.

In a majority of patients, PF4/vaccine complex formation and the vaccine-stimulated proinflammatory milieu appear to trigger a B cell response, resulting in the formation of high-avidity anti-PF4 antibodies [95]. The pathogenic anti-PF4 antibodies can induce platelet activation and formation of neutrophil extracellular traps, driving thrombosis and thrombocytopenia in VITT. Platelet activation responses measured by antibodies in VITT patients are P-selectin and phosphatidylserine expression, with only part a heparin dependency [96,97,98]. This implies that VITT can be considered to be a HIT-like syndrome in some patients with PF4-dependent neo-epitopes. At the time of writing, the primary treatment option in VITT is therapeutic anticoagulation with non-heparins and intravenous immunoglobulins.

## 8. Platelets in Thrombotic Thrombocytopenia Purpura (TTP)

TTP is a life-threatening occlusive disorder of the microcirculation. It is caused by the inability of cleavage of endothelial-derived ultra-large multimers of VWF, due to a deficiency of the proteolytic enzyme ADAMTS13 (a disintegrin and metalloproteinase with thrombospondin motifs 13) [99,100]. TTP patients develop platelet microaggregates, which can block small arterioles. The pathophysiology of ADAMTS13 deficiency is straightforward, given that VWF is the only physiological substrate for ADAMTS13 in plasma [101]. Once the ADAMTS13 activity is impaired, the ultra-large VWF multimers spontaneously bind to platelets, cause GPIb-V-IX-dependent platelet agglutination and subsequent platelet activation [102]. This formation of VWF-rich platelet microthrombi is a hallmark of TTP. Especially in the microcirculation, microthrombi grow by attracting other platelets; consequences are thrombocytopenia and the destruction of (trapped) red blood cells, which in turn leads to a hemolytic anemia with fragmented red blood cells (schistocytes) [103].

The defect in ADAMTS13 can have a genetic (congenital) or an acquired background. Hereditary TTP, or Upshaw-Schulman syndrome, is commonly diagnosed in children, whereas acquired TTP is mostly recognized in adults [104]. Congenital ADAMTS13 deficiency is an extremely rare heterogeneous disorder, although >200 unique ADAMTS13 mutations are described [105]. Acquired TTP results from the development of auto-antibodies against ADAMTS13, which often act as enzyme inhibitors (demonstrated by Bethesda-like assays) [106]. However, some TTP patients display non-inhibitory anti-ADAMTS13 auto-antibodies, which accelerate the clearance of ADAMTS13 from plasma. It is worth noting that moreover ADAMTS13 deficiency, also rare gain-of-function mutations in the A1 domain of VWF or in platelet GPIbα can result enhanced VWF-GPIbα interaction, even leading to thrombocytopenia (specific forms of von Willebrand disease) [107,108].

Patients with hereditary TTP can have symptoms starting from birth, but some patients remain asymptomatic for decades. This implies that a deficiency of ADAMTS13 alone is not sufficient to induce the clinical syndrome. Regarding acquired TTP, several determinants are considered to be additional risk factors, such as female sex, black ethnicity, HLA isoforms, obesity and high plasma VWF [109]. Acute TTP can be fatal when not adequately treated, e.g., by plasma exchange. For hereditary TTP, plasma infusion is currently the only option. In the future, recombinant ADAMTS13 (rhADAMTS13) products may become available for TTP treatment [109]. So far, gene therapy has only been successful in ADAMTS13 knockout mice, whereas effective translation to human use is lacking [110].

## 9. Platelets in Hemolysis, Elevated Liver Enzymes and Low Platelet (HELLP) Syndrome

The HELLP syndrome is a life-threatening complication for both mother and fetus, which occurs in 0.2–0.8% of pregnancies [111]. Similar to TTP, HELLP syndrome belongs to the thrombotic microangiopathy disorders, where the low platelet count usually results from consumption of circulating platelets in the form of platelet–fibrin thrombi [112].

Evidence is present that the level of active VWF is up-regulated and the metalloproteinase ADAMTS13 is decreased in HELLP syndrome, which contribute to the formation of intravascular microthrombi [113,114]. The local damage of endothelium releases ultra-large VWF multimers, which agglutinate and activate platelets, ultimately depleting the platelet count [115]. In addition, the coagulation cascade becomes activated on the damaged endothelium. Activated platelets release sCD40L, thromboxane A_2_ and serotonin, causing vasospasm and platelet aggregation, thus further promoting the endothelial damage [116]. Studies have also shown that an altered complement system can contribute to HELLP syndrome. This concerned mutations in genes encoding for proteins in the alternative complement pathway (factor H and I) [117]. In particular, lower levels of complement factor H were correlated with a reduced platelet count [118]. Additionally, several studies with patients with HELLP syndrome point to possible roles of the *F5* and *HELLPAR* (*LINC*) genes ( Supplementary Table S1) [119,120]. However, the correlation of these mutations in HELLP syndrome with platelet activation aspects requires further study.

## 10. Conclusions and Perspective

Platelets, the main players of hemostasis and thrombosis, have multiple regulating properties linking immune, complement, and coagulation systems. It is becoming apparent now that platelet activation can act as a biomarker of multiple IIT diseases. Several circulating substances, such as auto-antibodies, immune complexes, VWF, DAMPs, complement factors and extracellular vesicles, can trigger a sustained type of platelet activation. There possibly is a central role of the platelet activation via the FcγRIIA receptors, but to which extent the various auto-antibodies and larger immune complexes cause the same degree of receptor dimerization and clustering—and hence signaling strength—is still unclear. The activated platelets in turn modulate the function of the innate and adaptive immune system by releasing multiple pro-inflammatory and immune mediators or through direct cellular interactions with immune cells, such as B and T cells. It would be worth to systematically investigate the variety of platelet derived biomarkers for diagnosis, prognosis and successful treatment of IIT diseases reviewed in this paper. On the other hand, the roles of platelets and of platelet activation processes in these autoimmune diseases are still only partly understood. However, the research should not be limited to distinct platelet activation processes or distinct factors released by platelets. Additionally, the interactions between platelets with other immune cells such as B and T cells, macrophages and leukocytes require further clarification. We foresee that better understanding of the role of platelets will reveal new therapeutic options in the future.

## Data Availability

All background data are provided in the supplemental datafile.

## Supplementary Materials

The following are available online at https://www.mdpi.com/article/10.3390/cells10123386/s1, Sun Supplemental Datafile with Tables S1 and S2.
